# Performance, biochemical and haematological responses, and relative organ weights of laying hens fed diets supplemented with prebiotic, probiotic and synbiotic

**DOI:** 10.1186/s12917-017-1160-y

**Published:** 2017-08-17

**Authors:** Shirley Gee Hoon Tang, Chin Chin Sieo, Kalavathy Ramasamy, Wan Zuhainis Saad, Hee Kum Wong, Yin Wan Ho

**Affiliations:** 1 Department of Applied Sciences, Faculty of Science and Technology, Nilai University, No. 1, Persiaran Universiti, Putra Nilai, 71800 Nilai, Negeri Sembilan Malaysia; 2 Laboratory of Vaccines and Immunotherapeutics, Institute of Bioscience, Universiti Putra Malaysia, 43400 UPM, Serdang, Selangor Darul Ehsan Malaysia; 3 Faculty of Biotechnology and Biomolecular Sciences, Universiti Putra Malaysia, 43400 UPM, Serdang, Selangor Darul Ehsan Malaysia; 4 Collaborative Drug Discovery Research (CDDR) Group, Faculty of Pharmacy, Universiti Teknologi MARA (UiTM), 42300 Bandar Puncak Alam, Selangor Darul Ehsan, Malaysia; 5 Strategic Livestock Research Centre, Malaysian Agricultural Research and Development Institute (MARDI), Persiaran MARDI-UPM, 43400 Serdang, Selangor Darul Ehsan Malaysia

**Keywords:** Prebiotic, Probiotic, Synbiotic, Laying hen, Performance, Biochemistry, Haematology, Organ weight

## Abstract

**Background:**

The increasing trend of ban on the use of antibiotic growth promoters (AGPs) across the globe in the poultry industry has led to a growing need for alternatives to AGPs. Prebiotic, probiotic and their combination as a synbiotic have been considered as potential alternatives. This study aimed to investigate the effects of a prebiotic (isomaltooligosaccharide, IMO), a probiotic (PrimaLac®), and their combination (synbiotic) on hen performance, biochemical and haematological responses, and relative organ weights from 20 to 52 weeks of age.

**Results:**

Supplementation of 1% IMO (PRE), 0.1% PrimaLac® (PRO) and 1% IMO + 0.1% PrimaLac® (SYN) improved (*P* < 0.05) feed intake and egg production at 20–36 weeks of age; body weight gain, feed conversion ratio and egg mass at 20–36 and 20–52 weeks of age; and egg weight at 20–36, 37–52 and 20–52 weeks of age. Compared to control-fed hens at 20–36 weeks of age, PRO- and SYN-fed hens produced less (*P* < 0.05) small size eggs while SYN-fed hens produced more large size eggs. From 37 to 52 weeks of age, PRE-, PRO- or SYN-fed hens produced less (*P* < 0.05) medium size eggs, and more large and extra-large size eggs. PRE, PRO or SYN supplementation decreased (*P* < 0.05) the serum total cholesterol at 36 weeks of age, and serum low-density lipoprotein (LDL) cholesterol, alanine aminotransferase (ALT) and alkaline phosphatase (ALP) at 36 and 52 weeks of age. At 36 and 52 weeks of age, supplementation of PRE, PRO or SYN increased (*P* < 0.05) lymphocyte percentage and decreased (*P* < 0.05) heterophil percentage, leading to a lower heterophil to lymphocyte (H/L) ratio. No significant differences were observed in the relative weights of the heart, liver, ovary, pancreas and spleen of all dietary treatment groups.

**Conclusions:**

Supplementation of PRE, PRO or SYN improved performance, serum total cholesterol, LDL cholesterol, ALT, ALP and H/L ratio of hens from 20 to 52 weeks of age. These results demonstrated the use of PRE, PRO and SYN as alternative feed additives to AGPs for improving the health and productivity of hens, while PRO is the best for commercial layer production to yield maximum profit.

## Background

The imprudent use of antibiotic growth promoters (AGPs) in poultry production has led to the development of antibiotic-resistant bacteria and the accumulation of antibiotic residues in poultry products, which can pose a threat to consumers. Hence, many countries have begun to severely prohibit the use of AGPs in poultry production. Probiotics and prebiotics can be considered as natural feed additives which may be able to provide an effect similar to that of AGPs. The supplementation of probiotics in poultry feeds has been reported to improve growth performance [1–10], nutrient retention [11, 12], caecal microbial balance [2, 8, 11] and immune response [4, 13], and lower cholesterol levels of chickens [3, 5–7, 10, 13]. Intake of prebiotics can confer health benefits to poultry, such as modulating the colonic microbiota by increasing the number of specific probiotic bacteria, including lactobacilli and bifidobacteria [8]. Furthermore, prebiotics intake helps in reducing pathogenic bacteria by mimicking their attachment sites on the intestinal mucosa [14], enhancing mineral absorption [15], and reducing serum cholesterol level [16]. A combination of probiotic and prebiotic is often referred to as a synbiotic [17]. A synbiotic product has the potential synergistic effect of promoting the proliferation of existing strains of beneficial bacteria in the colon as well as improving the survival and growth of newly added probiotic strains [17].

Most of the studies on the beneficial effects of probiotics [1–8, 11], prebiotics [18, 19] and synbiotics [1, 8, 15] on chickens focused on broiler chickens. Studies on the effects of these supplements, particularly prebiotics and synbiotics, on laying hens were comparatively less. A study on prebiotic fructooligosaccharide (FOS) by Li et al. [16] showed that feed consumption, feed conversion ratio (FCR) and egg production of laying hens were improved when FOS was added to the diets. Chen et al. [20] also reported that prebiotic oligosaccharides, such as inulin and oligofructose, improved the egg production and FCR of White Leghorn layers. Similarly, Abdelqader et al. [21] found that supplementation of inulin, *Bacillus subtilis* and a combination of both improved the production performance, eggshell quality and microflora composition of laying hens. A significant reduction in egg yolk cholesterol of hens fed with different concentrations of prebiotic inulin was reported by Shang et al. [22]. However, Mohebbifar et al. [23] observed that the production performance, egg quality and blood parameters of the laying hens supplemented with probiotics (PrimaLac®, A-Max® and Yeasture®) and a prebiotic (Fermacto®) were not significantly improved.

Recently, we have shown that a prebiotic oligosaccharide, isomaltooligosaccharide (IMO), and its combination with a probiotic as a synbiotic, improved growth performance, feed efficiency and caecal microbial populations of broiler chickens [8]. However, it is not known whether the prebiotic IMO and its combination with a probiotic as a synbiotic have similar beneficial effects on laying hens. Thus, the present study was undertaken to evaluate the effects of IMO, administered independently and in combination with a probiotic (PrimaLac®) as a synbiotic, on the performance, biochemical and haematological responses, and relative organ weights of laying hens from 20 to 52 weeks of age. This is the first study of the dietary effects of the prebiotic, IMO, and its synbiotic on laying hens. The individual effect of the probiotic, PrimaLac®, was also studied to compare it with that of the synbiotic for determining the synergistic effect of the synbiotic.

## Methods

### Prebiotic and probiotic

The prebiotic IMO (Wako, Osaka, Japan) was mixed with the basal diet (mash form) daily at feeding time. The prebiotic IMO contained ≥90% high quality and pure IMO which was produced by the enzymatic conversion of starch (≥45% of isomaltose, isomaltotriose and panose) (Wako, Osaka, Japan). The starch sources were from corn and tapioca. The prebiotic IMO was purchased in a white fine granular powder form. The probiotic, PrimaLac® (Star Labs, Clarksdale, USA), was a lyophilized mix containing 1 × 10^9^ cfu/g of *Lactobacillus acidophilus*, *Lactobacillus casei*, *Bifidobacterium bifidum*, *Streptococcus faecium* and *Aspergillus oryzae*. IMO was mixed singly or in combination with PrimaLac® (as a synbiotic) into the basal feed each day.

### Birds, housing and dietary treatments

A total of 160 beak-trimmed Hisex Brown pullets, aged 16 weeks with a mean body weight (± standard deviation, SD) of 1318.33 ± 105.64 g, obtained from a local commercial layer farm, were used in the experiment. The pullets were raised under open housing and kept in individual wire layer cages (31 cm width × 51.5 cm length × 34 cm height). The pullets were randomised into four dietary treatment groups. Each dietary treatment group had 40 pullets (4 replicates, with 10 pullets per replicate). The replicates were designated as the experimental units and were randomised with respect to the dietary treatments. Sample size calculation was performed using G*Power Software, version 3.1.9.2. [24]. The required total sample size based on the software was 144 pullets (36 pullets per treatment, 4 replicates with 9 pullets per replicate). 10% attrition was expected, hence, the actual sample size required for the study is 160 pullets using the following formula: Corrected sample size = Sample size/[1-(% attrition/100)], as reported by Charan and Kantharia [25].

The four dietary treatments were: (1) basal diet (control), (2) basal diet +1% IMO (PRE), (3) basal diet +0.1% PrimaLac® (PRO) and (4) basal diet +1% IMO + 0.1% PrimaLac® (SYN). The basal diet (antibiotic-free) was a corn-soybean diet formulated to meet the nutrient requirements of laying hens according to the National Research Council (NRC) [26]. The composition of the basal diet is shown in Table 1. The experimental period was 32 weeks, from 20 to 52 weeks of age. Diets were fed ad libitum in a mash form and added to the feeder daily at 0900 h. Water was provided in continuous flow nipple drinkers throughout the experimental period. The pullets were given 4 weeks to acclimatise to the dietary treatments. The four dietary treatments were coded and the identity of treatments was blinded to all investigators involved in the feeding trial. The temperature and humidity of the surrounding environment were measured daily using a thermohydrometer. All animals’ management and sampling procedures in this study were conducted according to the guidelines of the Research Policy on Animal Ethics and Welfare of the Universiti Putra Malaysia, and the Guide for the Care and Use of Agricultural Animals in Agricultural Research and Teaching [27]. This manuscript was prepared in compliance with the ARRIVE (Animal Research: Reporting of In Vivo Experiments) Guidelines Checklist for animal in vivo experiments [28].

**Table 1:** Composition of the basal diet

Ingredient (%)	Amount
Corn	57.78
Soybean meal	28.36
Limestone	9.67
Dicalcium phosphate	1.51
Wheat middlings	1.00
Palm oil	1.00
Common salt	0.33
DL-Methionine	0.17
Choline Cl-70%	0.06
Mineral premix^a^	0.10
Vitamin premix^b^	0.03
Total	100.00
*Calculated analysis*	
Crude protein	17.40
Crude fat	3.72
Crude fibre	2.62
Calcium	3.91
Available phosphorus	0.42
Lysine	0.93
Methionine	0.42
Lysine + cysteine	0.71
Metabolizable energy (MJ/kg)	11.70

^a^Mineral premix (per kg diet): 80 mg iron, 100 mg manganese, 15 mg copper, 80 mg zinc, 1 mg iodine, 0.2 mg selenium, 0.25 mg cobalt, 4 mg potassium, 0.6 mg magnesium and 1.5 mg sodium

^b^Vitamin premix (per kg diet): 15,000 IU vitamin A, 3000 IU vitamin D_3_, 22.5 mg vitamin E, 6 mg vitamin K_3_, 3 mg vitamin B_1_, 9 mg vitamin B_2_, 6 mg vitamin B_6_, 0.03 mg vitamin B_12_, 18 mg calcium D-panthothenate, 60 mg niacin, 1.5 mg folic acid, and 0.075 mg biotin

### Hen performance and sampling procedures

Eggs from 160 hens (40 hens per dietary treatment group, 4 replicates with 10 hens per replicate) were collected and weighed daily. Feed intake, FCR, hen-day egg production and egg mass were determined on a weekly basis. All eggs laid for 3 consecutive days at the end of each 4-week period, from 20 to 52 weeks of age, were collected for the determination of egg size. The size distribution of small (42.50–49.59 g), medium (49.60–56.69 g), large (56.70–63.78 g), extra-large (63.79–70.87 g) and jumbo size eggs (>70.88 g) was determined based on specified weights according to the United States Department of Agriculture (USDA) [29]. The percentage of eggs with specific size was calculated as follows:

$$ \mathrm{Percentage}\  \mathrm{of}\  \mathrm{eggs}\  \mathrm{with}\  \mathrm{specific}\  \mathrm{size}\ \left(\%\right)=\frac{\mathrm{Total}\  \mathrm{number}\  \mathrm{of}\  \mathrm{eggs}\  \mathrm{with}\  \mathrm{specific}\ \mathrm{egg}\ \mathrm{size}\  \mathrm{during}\  \mathrm{the}\  \mathrm{period}\ }{\mathrm{Total}\  \mathrm{number}\  \mathrm{of}\  \mathrm{eggs}\  \mathrm{laid}\ \mathrm{on}\ \mathrm{the}\  \mathrm{same}\  \mathrm{period}}\times 100\% $$

All the hens were weighed at the beginning of the experiment (20 weeks of age) and at the end of the experiment (36 and 52 weeks of age) for calculating the body weight gain. Feed cost for each dietary treatment per hen per day for the whole experimental period was calculated based on the feed intake, and total expenses for basal feed, probiotic PrimaLac® and prebiotic IMO. At 36 and 52 weeks of age, 12 hens from each treatment group (4 replicates, 3 hens from each replicate) were randomly selected, weighed and killed humanely for the analysis of biochemical and haematological parameters and the determination of relative organ weights. The collected samples were coded and analysed by the same investigators, who were blinded to dietary treatment group assignments.

### Analysis of biochemical and haematological parameters

Approximately 10 ml and 5 ml of blood were taken from the jugular vein of each hen and were collected in BD Vacutainer® Plus Plastic Serum Tubes (Becton Dickinson, New Jersey, USA) and BD Vacutainer® Plus Plastic K_2_EDTA Tubes (Becton Dickinson, New Jersey, USA), respectively. Blood samples collected in the BD Vacutainer® Plus Plastic Serum Tubes were centrifuged at 2500×*g* for 10 min, and the serum was separated for serum biochemical analysis using an automatic clinical chemistry analyser (Hitachi, Minato-ku, Japan). The serum biochemical parameters analysed were alanine aminotransferase (ALT), alkaline phosphatase (ALP), aspartate aminotransferase (AST), gamma-glutamyl transpeptidase (GGT), creatinine kinase (CK), triglyceride, total cholesterol, low-density lipoprotein (LDL) cholesterol, high-density lipoprotein (HDL) cholesterol, total protein, uric acid, glucose, calcium, phosphorus, sodium, potassium and chloride. Blood samples collected in the BD Vacutainer® Plus Plastic K_2_EDTA Tubes were used for haematological analysis. An automated haematological analyser (CELL-DYN 3700 Abbott Diagnostics, USA) was used for the following haematological parameters: red blood cell count (RBC), haemoglobin (Hb), packed cell volume (PCV), mean corpuscular volume (MCV), mean corpuscular haemoglobin concentration (MCHC) and thrombocyte count. A differential leukocytic count (percentage of total) for heterophils, lymphocytes, monocytes, eosinophils and basophils was performed by manual examination of the blood smears stained with Wright’s stain (Sigma Chemical Co., Missouri, USA). Differential leukocyte count was obtained based on 200 leukocytes. The heterophil to lymphocyte ratio (H/L) was calculated.

### Determination of relative organ weights

The weights of the hens were recorded prior to euthanise. The carcasses were opened immediately, and the heart, liver, ovary, pancreas and spleen were removed and weighed. The relative organ weight was calculated as follows:

$$ \mathrm{Relativeorganweight}=\frac{\mathrm{Weightoforgan}\left(\mathrm{g}\right)}{\mathrm{Bodyweight}\left(\mathrm{g}\right)}\times 100\% $$

### Statistical analysis

Statistical analysis of the results was performed using SPSS for Windows version 16.0 software (SPSS Inc., Chicago, USA). The assumption of normality was tested using the Kolmogorov-Smirnov test and visual assessment of Quantile-Quantile (Q-Q) plots of model residuals. Homogeneity of variance was assessed using Levene’s test. The normality and equality of variance assumptions were not violated, thus, all data were subjected to One-Way Between Groups Analysis of Variance (ANOVA). Duncan’s Multiple Range Test was carried out for post-hoc comparison to detect the differences between dietary treatments. All differences were considered significant at *P* < 0.05. For all analyses, the replicate was considered as the experimental unit. The results were presented as mean ± SD in all tables (Tables 2 to 5).

**Table 2:** Effects of dietary treatments on body weight gain, feed intake, feed conversion ratio, egg production, egg weight, egg mass and feed cost of laying hens from 20 to 52 weeks of age

Parameter	Dietary treatment			
	Control	PRE	PRO	SYN
Body weight gain (g)				
20–36 weeks	369.58 ± 71.36^b^	473.54 ± 62.70^a^	497.08 ± 38.11^a^	466.67 ± 45.55^a^
37–52 weeks	492.92 ± 49.83	532.09 ± 58.11	523.34 ± 36.01	508.54 ± 42.36
20–52 weeks	424.37 ± 41.43^b^	502.81 ± 41.72^a^	510.21 ± 35.67^a^	487.61 ± 32.16^a^
Feed intake (g/hen/day)				
20–36 weeks	95.82 ± 1.61^b^	100.46 ± 1.19^a^	99.37 ± 1.69^a^	99.85 ± 1.00^a^
37–52 weeks	108.77 ± 2.57	112.11 ± 2.61	109.60 ± 1.69	111.12 ± 2.48
20–52 weeks	102.29 ± 0.89	103.28 ± 4.73	104.49 ± 1.65	105.49 ± 1.60
Feed conversion ratio				
20–36 weeks	2.55 ± 0.09^a^	2.39 ± 0.07^b^	2.32 ± 0.08^b^	2.33 ± 0.11^b^
37–52 weeks	2.18 ± 0.17	2.06 ± 0.08	2.02 ± 0.08	2.10 ± 0.07
20–52 weeks	2.34 ± 0.13^a^	2.14 ± 0.07^b^	2.15 ± 0.06^b^	2.20 ± 0.06^b^
Egg production (%)				
20–36 weeks	69.29 ± 2.86^b^	75.77 ± 3.62^a^	76.51 ± 2.44^a^	77.00 ± 5.49^a^
37–52 weeks	85.18 ± 6.37	89.91 ± 2.48	89.62 ± 4.69	87.77 ± 3.61
20–52 weeks	78.04 ± 2.83	82.84 ± 1.80	83.06 ± 3.12	82.38 ± 2.90
Egg weight (g)				
20–36 weeks	53.62 ± 0.27^b^	54.82 ± 1.03^a^	55.07 ± 0.80^a^	55.21 ± 0.67^a^
37–52 weeks	58.75 ± 0.84^b^	60.30 ± 0.76^a^	60.54 ± 0.73^a^	60.25 ± 0.17^a^
20–52 weeks	56.19 ± 0.46^b^	57.56 ± 0.86^a^	57.81 ± 0.70^a^	57.73 ± 0.42^a^
Egg mass (g)				
20–36 weeks	37.68 ± 1.34^b^	42.08 ± 1.05^a^	42.88 ± 1.83^a^	43.06 ± 2.95^a^
37–52 weeks	50.08 ± 2.06	54.42 ± 1.46	54.25 ± 2.66	52.88 ± 2.18
20–52 weeks	43.88 ± 2.57^b^	48.25 ± 0.91^a^	48.57 ± 1.91^a^	47.97 ± 1.60^a^
Feed cost (hen/day)(RM)	0.25 ± 0.003^b^	0.49 ± 0.002^a^	0.26 ± 0.004^b^	0.51 ± 0.01^a^

Data represent mean ± SD of four replicates of 10 hens each

^a,b^Means within a row with different superscripts differ significantly (*P* < 0.05)

Control, basal diet; PRE, basal diet +1% IMO; PRO, basal diet +0.1% PrimaLac®; SYN, basal diet +1% IMO + 0.1% PrimaLac®

## Results

### Performance

The results presented in Table 2 show the effects of the prebiotic (PRE), probiotic (PRO) and synbiotic (SYN) on body weight gain, feed intake, FCR, hen-day egg production, egg weight, egg mass and feed cost of laying hens from 20 to 52 weeks of age. At the early stage of laying period, from 20 to 36 weeks of age, the feed intake and egg production of the PRE-, PRO- or SYN-fed hens were significantly (*P* < 0.05) increased compared to those of the control hens. However, from 37 to 52 and 20 to 52 weeks of age, there were no significant differences in the feed intake and egg production among the treatments. The body weight gain, FCR and egg mass of hens fed PRE, PRO or SYN diet were significantly (*P* < 0.05) improved from 20 to 36 and 20 to 52 weeks of age, but not from 37 to 52 weeks of age, compared to those fed control diet. The egg weight of hens supplemented with PRE, PRO or SYN was significantly (*P* < 0.05) heavier than that of the control hens from 20 to 36, 37 to 52, and 20 to 52 weeks of age. Hens supplemented with PRO diet had significantly (*P* < 0.05) lower feed cost than hens from PRE- and SYN-supplemented groups. Meanwhile, the egg production and egg weight of PRO-supplemented hens were comparable to those of PRE- and SYN-supplemented hens.

The effects of the prebiotic, probiotic and synbiotic on egg size distribution of hens from 20 to 36 and 37 to 52 weeks of age are shown in Table 3. During the early phase of the egg laying period, from 20 to 36 weeks of age, hens receiving PRO or SYN diet produced a significantly (*P* < 0.05) lower percentage of small size eggs (7.32 ± 2.06% and 7.55 ± 2.38%, respectively) compared to that of the control hens (12.69 ± 1.27%). During this laying period, PRE-, PRO- or SYN-fed hens produced a significantly (*P* < 0.05) lower percentage of medium size eggs (46.55 ± 2.63%, 43.42 ± 3.79% and 38.13 ± 3.24%, respectively) than that of the control-fed hens (52.31 ± 5.01%), with SYN-fed hens showing the lowest percentage of medium size eggs. However, hens receiving the SYN diet produced a significantly (*P* < 0.05) higher percentage of large size eggs (46.62 ± 5.31%) during this period, compared to those receiving other dietary treatments (30.31 ± 7.50% - 41.96 ± 5.15%). There were no significant differences in the percentages of extra-large size eggs among all dietary treatment groups from 20 to 36 weeks of age. From 37 to 52 weeks of age, there was an increase in the percentages of large and extra-large size eggs in all dietary treatment groups. During this laying period, hens supplemented with PRE, PRO or SYN had a significantly (*P* < 0.05) lower percentage of medium size eggs (16.74 ± 3.19%, 13.32 ± 1.68% and 13.81 ± 1.73%, respectively) and a higher percentage of large (60.23 ± 4.70%, 63.57 ± 3.30% and 62.68 ± 4.91%, respectively) and extra-large size eggs (20.20 ± 2.61%, 20.41 ± 3.97% and 20.66 ± 3.50%, respectively), compared to hens offered the control diet (medium size eggs, 28.13 ± 4.50%; large size eggs, 57.17 ± 5.43% and extra-large size eggs, 12.67 ± 3.24%). No significant difference was observed in the percentages of jumbo size eggs among the dietary treatments.

**Table 3:** Effects of dietary treatments on egg size of laying hens from 20 to 52 weeks of age

Percentage of eggs with specific egg size (%)	Dietary treatment			
	Control	PRE	PRO	SYN
20–36 weeks of age				
XL	4.69 ± 0.96	7.60 ± 3.23	7.30 ± 1.65	7.70 ± 3.82
L	30.31 ± 7.50^b^	34.85 ± 7.08^b^	41.96 ± 5.15^b^	46.62 ± 5.31^a^
M	52.31 ± 5.01^a^	46.55 ± 2.63^b^	43.42 ± 3.79^b^	38.13 ± 3.24^c^
S	12.69 ± 1.27^a^	11.00 ± 0.11^ab^	7.32 ± 2.06^c^	7.55 ± 2.38^bc^
37–52 weeks of age				
Jumbo	2.04 ± 0.59	2.83 ± 0.44	2.69 ± 0.76	2.85 ± 0.59
XL	12.67 ± 3.24^b^	20.20 ± 2.61^a^	20.41 ± 3.97^a^	20.66 ± 3.50^a^
L	57.17 ± 5.43^b^	60.23 ± 4.70^a^	63.57 ± 3.30^a^	62.68 ± 4.91^a^
M	28.13 ± 4.50^a^	16.74 ± 3.19^b^	13.32 ± 1.68^b^	13.81 ± 1.73^b^

Data represent mean ± SD of four replicates of 10 hens each

^a-c^Means within a row with different superscripts differ significantly (*P* < 0.05)

Control, basal diet; PRE, basal diet +1% IMO; PRO, basal diet +0.1% PrimaLac®; SYN, basal diet +1% IMO + 0.1% PrimaLac®

Jumbo (>70.88 g); XL, Extra Large (63.79–70.87 g); L, Large (56.70–63.78 g); M, Medium (49.60–56.69 g); S, Small (42.50–49.59 g)

The results obtained from the present study indicated that the supplementation of PRE, PRO or SYN in the diets of laying hens influenced a shift from small to large size eggs between 20 to 36 weeks of age and a shift from medium to large and extra-large size eggs between 37 to 52 weeks of age.

### Biochemical and haematological parameters

The effects of dietary treatments on serum biochemical parameters of laying hens at 36 and 52 weeks of age are summarised in Table 4. Significantly (*P* < 0.05) lower levels of serum total cholesterol were observed in the PRE-, PRO- or SYN-supplemented group compared to that of the control group at 36 weeks of age, but not at 52 weeks of age. All the supplemented dietary treatment groups (PRE, PRO and SYN) had significantly (*P* < 0.05) decreased serum LDL cholesterol levels at 36 and 52 weeks of age compared to that of the control group. However, there were no significant differences in the levels of serum HDL cholesterol and triglycerides among all dietary treatment groups at 36 and 52 weeks of age. The levels of serum ALT and ALP were significantly (*P* < 0.05) lower in hens fed with PRE, PRO or SYN diet compared to those in hens fed with the control diet at 36 and 52 weeks of age. No significant differences (*P* > 0.05) were observed in the levels of serum AST, GGT, CK, total protein, uric acid, calcium, phosphorus, sodium, potassium, chloride and glucose among the dietary treatment groups at 36 and 52 weeks of age.

**Table 4:** Effects of dietary treatments on serum biochemical parameters of laying hens at 36 and 52 weeks of age

Serum biochemical parameters	Hen age (weeks)							
	36 weeks of age				36 weeks of age			
	Control	PRE	PRO	SYN	Control	PRE	PRO	SYN
Total cholesterol (mmol/L)	3.72 ± 0.35^a^	3.11 ± 0.22^b^	2.87 ± 0.44^b^	2.92 ± 0.40^b^	2.80 ± 0.55	2.39 ± 0.28	2.69 ± 0.33	2.46 ± 0.10
LDL (mmol/L)	0.13 ± 0.05^a^	0.08 ± 0.02^b^	0.07 ± 0.04^b^	0.06 ± 0.02^b^	0.09 ± 0.03^a^	0.06 ± 0.01^b^	0.04 ± 0.01^b^	0.05 ± 0.01^b^
HDL (mmol/L)	0.21 ± 0.04	0.24 ± 0.04	0.25 ± 0.03	0.26 ± 0.05	0.26 ± 0.06	0.29 ± 0.04	0.31 ± 0.05	0.38 ± 0.04
Triglyceride (mmol/L)	14.15 ± 3.67	13.95 ± 2.12	12.49 ± 3.38	13.51 ± 1.65	14.54 ± 3.33	12.83 ± 2.38	14.17 ± 2.23	12.73 ± 0.97
ALT (U/L)	25.21 ± 7.77^a^	8.34 ± 1.58^b^	5.19 ± 0.69^b^	4.05 ± 1.32^b^	12.38 ± 3.18^a^	9.81 ± 2.11^b^	6.55 ± 0.93^b^	8.39 ± 1.12^b^
ALP (U/L)	407.79 ± 42.31^a^	285.58 ± 31.91^bc^	213.88 ± 24.07^c^	314.34 ± 32.86^b^	288.74 ± 39.80^a^	207.46 ± 34.75^b^	215.14 ± 22.34^b^	211.50 ± 21.52^b^
AST (U/L)	272.98 ± 31.03	238.20 ± 11.42	240.63 ± 24.07	249.00 ± 34.17	245.80 ± 12.74	244.95 ± 11.84	233.85 ± 17.18	242.48 ± 10.86
GGT (U/L)	39.25 ± 4.65	27.50 ± 9.78	27.50 ± 2.87	28.50 ± 3.87	18.75 ± 3.78	16.17 ± 4.01	15.75 ± 4.09	18.17 ± 1.85
CK (U/L)	4524.50 ± 505.30	4088.00 ± 526.83	3992.50 ± 700.14	4263.00 ± 737.99	4504.50 ± 595.81	3979.50 ± 425.55	4080.00 ± 639.79	3983.25 ± 350.15
Total protein (g/L)	58.20 ± 1.54	61.16 ± 2.95	57.68 ± 2.33	57.90 ± 3.64	50.76 ± 4.91	49.00 ± 3.48	49.33 ± 4.13	46.06 ± 1.01
Uric acid (μmol/L)	196.40 ± 27.13	193.64 ± 12.09	198.50 ± 17.84	190.67 ± 13.37	255.70 ± 11.90	209.78 ± 27.72	221.45 ± 14.73	244.93 ± 13.09
Ca (mmol/L)	5.93 ± 0.52	6.41 ± 0.12	6.17 ± 0.35	6.39 ± 0.26	4.37 ± 0.43	4.61 ± 0.25	4.56 ± 0.13	4.69 ± 0.20
P (mmol/L)	1.70 ± 0.26	1.86 ± 0.15	1.59 ± 0.16	1.70 ± 0.11	1.55 ± 0.19	1.73 ± 0.22	1.77 ± 0.20	1.74 ± 0.19
Na (mmol/L)	157.75 ± 1.06	160.36 ± 1.16	160.20 ± 3.95	157.19 ± 1.05	152.17 ± 1.05	156.79 ± 1.95	155.16 ± 0.40	155.34 ± 1.08
K (mmol/L)	4.85 ± 0.29	4.70 ± 0.26	4.45 ± 0.17	4.83 ± 0.53	5.00 ± 0.48	4.68 ± 0.29	5.00 ± 0.66	5.35 ± 0.38
Cl (mmol/L)	121.11 ± 1.91	123.00 ± 1.00	123.37 ± 4.62	121.78 ± 1.56	114.24 ± 2.16	119.02 ± 0.99	118.78 ± 0.50	116.13 ± 0.96
Glucose (mmol/L)	12.15 ± 0.62	12.13 ± 0.29	11.93 ± 0.29	12.30 ± 0.52	10.61 ± 0.49	11.19 ± 0.64	11.02 ± 0.38	11.14 ± 0.21

*LDL* low-density lipoprotein cholesterol, *HDL* high-density lipoprotein cholesterol, *ALT* alanine aminotransferase, *ALP* alkaline phosphatase, *AST* aspartate aminotransferase, *GGT* gamma-glutamyl transpeptidase, *CK* creatinine kinase, *Ca* calcium, *P* phosphorus, *Na* sodium, *K* potassium, *Cl* chloride

Data represent mean ± SD of four replicates of three hens each

^a-c^ Means within a row at a particular age with different superscripts differ significantly (*P* < 0.05)

Control, basal diet; PRE, basal diet +1% IMO; PRO, basal diet +0.1% PrimaLac®; SYN, basal diet +1% IMO + 0.1% PrimaLac®

Haematological parameters of hens fed with the four dietary treatments are shown in Table 5. Hens supplemented with PRE, PRO or SYN had a significantly (*P* < 0.05) higher lymphocyte percentage but lower heterophil percentage, resulting in a lower H/L ratio than that of the control hens at 36 and 52 weeks of age. No significant differences (*P* > 0.05) were found in the amounts of RBC, Hb, PCV, MCV, MCHC, WBC, thrombocyte, and percentages of monocytes, eosinophils and basophils among all dietary treatment groups at 36 and 52 weeks of age.

**Table 5:** Effects of dietary treatments on haematological parameters of laying hens at 36 and 52 weeks of age

Haematological parameters	Hen age (weeks)							
	36 weeks of age				52 weeks of age			
	Control	PRE	PRO	SYN	Control	PRE	PRO	SYN
Lymphocyte (%)	33.16 ± 1.55^b^	41.22 ± 0.70^a^	40.16 ± 1.13^a^	41.70 ± 1.25^a^	28.87 ± 0.79^b^	32.59 ± 2.23^a^	31.97 ± 2.45^a^	33.96 ± 1.94^a^
Heterophil (%)	58.18 ± 1.94^a^	49.75 ± 1.52^b^	49.91 ± 1.17^b^	47.34 ± 1.45^b^	60.87 ± 1.18^a^	56.79 ± 1.81^b^	57.74 ± 1.06^b^	55.46 ± 1.27^b^
H/L ratio	1.65 ± 0.17^a^	1.17 ± 0.06^b^	1.11 ± 0.03^b^	1.07 ± 0.15^b^	2.11 ± 0.09^a^	1.75 ± 0.18^b^	1.82 ± 0.17^b^	1.64 ± 0.14^b^
RBC (× 10^12^/L)	2.34 ± 0.13	2.38 ± 0.21	2.33 ± 0.15	2.49 ± 0.14	2.13 ± 0.07	2.14 ± 0.10	2.21 ± 0.05	2.22 ± 0.14
Hb (g/L)	118.56 ± 5.97	126.67 ± 6.66	115.50 ± 5.64	122.17 ± 2.26	112.00 ± 2.46	113.13 ± 1.75	112.92 ± 2.91	115.71 ± 1.70
PCV (L/L)	0.27 ± 0.01	0.27 ± 0.01	0.27 ± 0.01	0.27 ± 0.01	0.25 ± 0.02	0.23 ± 0.01	0.24 ± 0.01	0.26 ± 0.01
MCV (fL)	120.54 ± 1.78	126.50 ± 1.53	118.91 ± 2.60	120.42 ± 6.55	112.55 ± 1.22	113.42 ± 1.23	112.57 ± 1.65	115.73 ± 3.07
MCHC (g/L)	420.77 ± 18.77	444.01 ± 23.12	439.77 ± 6.49	440.17 ± 5.74	456.38 ± 5.32	470.98 ± 14.75	458.48 ± 6.54	460.35 ± 13.39
WBC (× 10^9^/L)	23.57 ± 2.19	23.92 ± 1.02	24.76 ± 1.78	24.57 ± 1.67	23.85 ± 3.87	24.88 ± 4.27	24.13 ± 4.70	25.28 ± 2.20
Thrombocyte (× 10^9^/L)	24.49 ± 2.50	27.58 ± 3.72	24.83 ± 1.25	28.07 ± 0.78	21.60 ± 2.44	24.73 ± 4.18	25.78 ± 2.49	25.32 ± 3.31
Monocyte (%)	3.50 ± 0.50	3.27 ± 0.75	3.67 ± 1.26	4.67 ± 0.58	4.03 ± 0.19	3.97 ± 0.62	3.81 ± 0.47	3.83 ± 0.53
Eosinophil (%)	2.27 ± 0.93	2.67 ± 0.76	3.00 ± 0.87	3.17 ± 0.76	3.20 ± 0.42	3.25 ± 0.46	3.38 ± 0.92	3.57 ± 0.54
Basophil (%)	2.90 ± 0.66	3.10 ± 0.53	3.27 ± 0.25	3.13 ± 0.65	3.04 ± 0.53	3.40 ± 0.44	3.10 ± 0.78	3.19 ± 0.55

*H/L* heterophil/lymphocyte, *RBC* red blood cell, *Hb* haemoglobin, *PCV* packed cell volume, *MCV* mean corpuscular volume, *MCHC* mean corpuscular haemoglobin concentration, *WBC* white blood cell

Data represent mean ± SD of four replicates of three hens each

^a,b^Means within a row at a particular age with different superscripts differ significantly (*P* < 0.05)

Control, basal diet; PRE, basal diet +1% IMO; PRO, basal diet +0.1% PrimaLac®; SYN, basal diet +1% IMO + 0.1% PrimaLac®

### Relative organ weights

There were no significant differences (*P* > 0.05) in the relative organ weights of the heart, liver, ovary, pancreas and spleen of the laying hens given different dietary treatments (data not shown).

## Discussion

The supplementation of PRE, PRO and SYN in the diets of laying hens had significantly improved the body weight gain, feed intake, FCR, egg production, egg weight, egg mass and egg size at 20–36, 37–52 and/or 20–52 weeks of age as compared to those of control hens. However, the improvements made by SYN were not significantly different from those made by PRE or PRO alone. This indicated that the inclusion of SYN did not provide a two-fold synergistic effect on the performance of laying hens. This result is in agreement with that of Abdelqader et al. [21]*,* where the supplementation of *Bacillus subtilis* and inulin did not show a synergistic effect on the productive performance of laying hens. Youssef et al. [30] also observed that there was no significant synergistic improvement in the performance of laying hens supplemented with synbiotic when compared to prebiotic and probiotic alone.

In the present study, the feed intake of laying hens was improved by the supplementation of PRE, PRO or SYN at 20–36 weeks of age. There are several studies in which the supplementation of prebiotics, probiotics or synbiotics in poultry feeds has improved the feed intake of broiler chickens and laying hens [31–33], and it has been suggested that the improved feed intake is most likely due to the ability of the probiotic and prebiotic to stimulate the appetite of laying hens [12, 32]. In addition, it may be associated with the improved body weight gain of the supplemented hens [32]. The results of the present study, in which there was an improvement in the body weight gain of the laying hens supplemented with PRE, PRO or SYN diet, lend support to the latter suggestion. However, there are some studies which reported that the addition of prebiotics, probiotics or synbiotics had no effect on the feed intake of laying hens or broiler chickens [8, 21, 23].

Supplementation of PRE, PRO or SYN improved the FCR of laying hens in the present study at 20–36 and 20–52 weeks of age. In a recent study, Abdelqader et al. [21] reported that the inclusion of *Bacillus subtilis* and inulin, individually or in a combination, in hen diets significantly improved FCR. Earlier, Kalavathy et al. [9] observed that the supplementation of a mixture of 12 *Lactobacillus* strains in hen diets significantly improved FCR from 20 to 35 weeks of age. Improvement in FCR was also found in hens supplemented with chicory oligofructose and inulin [20], FOS [16], and *L. acidophilus* [34]. The exact mechanism (s) underlying the beneficial effect of probiotics and prebiotics on the performance of chickens is not fully elucidated, but it is apparent that both probiotics and prebiotics function by manipulating the composition of intestinal microbiota. Several studies have demonstrated that the addition of probiotics [2, 8], prebiotics [8, 18] or synbiotics [8, 21] could regulate the intestinal microbial ecology and subsequently provide beneficial effects on the health and performance of the host animal. Huang et al. [35] suggested that an increase in nutrient digestion and absorption is the major mechanism responsible for the enhanced performance of chickens in response to prebiotic and probiotic supplementations. Recently, Meng et al. [36] showed that supplementation of chito-oligosaccharide increased dry matter and nitrogen digestibility and improved the growth performance of hens.

The results from the current study showed that the supplementation of PRE, PRO or SYN in hen diets improved egg production only during the early laying period, from 20 to 36 weeks of age. Significant improvements in egg production have also been reported in hens supplemented with a mixed culture of 12 *Lactobacillus* strains from 20 to 35 weeks of age [9], *L. sporogenes* from 25 to 40 weeks of age [13], *L. acidophilus* from 20 to 36 weeks of age [34], FOS from 26 to 42 weeks of age [16] and inulin from 29 to 39 weeks of age [37]. A study on hens fed with a commercial synbiotic, Biomin IMBO, also showed improved egg production of White Leghorn layers throughout a 7-week experimental period [38]. The authors suggested that the improvements in egg production of laying hens were due to the ability of probiotics and prebiotics to enhance nutrient digestibility and absorption in the gastrointestinal tract of hens.

Hens fed with PRE, PRO or SYN diet in this study produced significantly greater egg weight and egg mass than those of the control hens from 20 to 52 weeks of age. Similar significant improvements in egg weight were also observed in hens supplemented with PrimaLac® from 18 to 70 weeks of age [39], a multi-strain probiotic (consisting of 12 *Lactobacillus* strains) from 20 to 35 weeks of age [9], inulin and oligofructose from 57 to 61 weeks of age [20], and inulin from 29 to 39 weeks of age [37]. The improved egg weight obtained in this study and previous studies is most likely due to the ability of PRE-, PRO- and SYN-fed hens to perform well under stressful conditions. It has been reported that the egg weight of egg-type hens could be largely affected by environment factors, although egg weight is highly heritable in chickens [40]. Since the laying hens in this study were placed in an open environment, the effect of the prebiotic, probiotic and synbiotic on the stress management of hens was expected to be more obvious. Therefore, the ability of supplemented hens to produce eggs with increased weight shows that they have a higher immunity to stressful conditions. Some studies have shown that the addition of prebiotics and probiotics reduced stress in laying hens [41, 42]. Furthermore, Shini et al. [43] have provided some initial evidences that reproductive pathologies which often cause the decrease in egg production and egg weight could be reduced by the supplementation of probiotics in laying hens. Thus, it is not surprising that the increase of egg weight occurred in PRO-fed hens. As prebiotics function as feed for the intestinal health-promoting bacteria, a similar improvement in egg weight also occurred in PRE- and SYN-fed hens.

The inclusion of PRE, PRO and SYN in layer diets improved the egg size distribution by influencing a shift from small and medium to large and extra-large size eggs in the current study. Significant improvements in egg size have also been reported in hens supplemented with *Lactobacillus*-based probiotics in several studies conducted by Kalavathy et al. [9] and Nahashon et al. [12, 32]. Grimes et al. [44] observed a shift in the egg size profile from large to extra-large size eggs in Single Comb White Leghorn hens fed with a commercial prebiotic, Fermacto. Davis and Anderson [39] reported similar results in which Single Comb White Leghorn hens fed with PrimaLac® produced a significantly higher percentage of extra-large size eggs. The increased egg size of hens supplemented with prebiotic or probiotic may be associated with the improved retention of nitrogen and calcium [31, 32], and stimulation of appetite [12, 32] by the prebiotic and probiotic.

The results from the current study indicated that prebiotic, probiotic or synbiotic supplementation had a hypocholesterolaemic effect on laying hens. At 36 weeks of age, the serum total cholesterol was significantly reduced. Similar hypocholesterolaemic effects were also observed in the serum of hens fed with *L. sporogenes* [13], Probiolac [45], and oligofructose and inulin [46]. However, Mohammadian et al. [38] reported that the supplementation of a synbiotic (Biomin IMBO) in laying hens did not significantly lower the serum total cholesterol.

Although the serum LDL cholesterol levels of laying hens were reduced after PRE, PRO or SYN treatment at 36 and 52 weeks of age, no significant differences in serum HDL cholesterol and triglyceride levels were observed. Similar findings were reported by Zarei et al. [47] and Taherpour et al. [48] in laying hens and broiler chickens, respectively.

Results from this and previous studies indicated that prebiotics, probiotics and synbiotics have a hypocholesterolaemic effect on the host animal. However, the hypocholesterolaemic mechanism(s) of probiotics and prebiotics has not yet been fully elucidated. Several mechanisms have been proposed to explain the hypocholesterolaemic effects of probiotic: (1) assimilation of cholesterol by probiotics [49], (2) production of bile salt hydrolase (BSH) enzyme by probiotics, leading to greater excretion of faecal bile acids [50, 51], (3) conversion of cholesterol to coprostanol by cholesterol reductase, which is produced by probiotics [52] and (4) inhibition of hydroxymethylglutaryl coenzyme A (HMG CoA) reductase by probiotic, an enzyme which is involved in cholesterol-synthesising pathway [53].

The hypocholesterolaemic effect of prebiotics is not clearly understood. However, some studies have reported that the hypocholesterolaemic effect of prebiotics could be due to the production of short chain fatty acids (SCFA) from prebiotic fermentation by intestinal microorganisms, which mainly contained acetic, propionic and butyric acids [22, 54]. It has been proposed that SCFA could suppress hepatic cholesterol synthesis [54] and stimulate bile acid synthesis [55], which could lead to the lowering of plasma cholesterol levels. Furthermore, since prebiotics are the food source of probiotics, they stimulate the growth of probiotics, resulting in the increase in deconjugation and faecal excretion of bile acids [22]. These are indirect mechanisms through which prebiotics may exert their hypocholesterolaemic effects.

Most of the mechanisms proposed thus far are focused on the hypocholesterolaemic effects of probiotics or prebiotics individually, rather than the mechanisms involved in a synbiotic treatment. Liong et al. [56] suggested that synbiotic induced hypocholesterolaemic effect by altering the pathway of cholesteryl esters and lipoprotein transporters [very-low-density lipoprotein (VLDL), LDL and HDL].

The presence of serum enzymes and their quantity in the serum can provide some indications of the degree of organ or tissue damage. The serum concentration of liver enzymes, such as AST, ALT and GGT, can be used to evaluate avian hepatic function because their synthesis occurs in the liver [57]. In this study, results on the serum biochemical parameters showed that laying hens fed with PRE, PRO or SYN diet had lower activities of ALT and ALP at 36 and 52 weeks of age compared to hens fed with control diet. There is a paucity of literature on the dietary effects of prebiotics, probiotics and synbiotics on enzyme activities in laying hens. Vahdatpour et al. [58] found that the activities of ALT and ALP were lower in female Japanese quails fed with a prebiotic (Fermacto®), probiotic (Protexin®) or synbiotic (combination of Fermacto® and Protexin®) than in control quails. A similar result was reported by Salarmoini and Fooladi [59], in which broiler chickens supplemented with a probiotic (Bioplus2) or fermented milk containing *L. acidophilus* exhibited lower levels of serum ALT and ALP than those of the control broiler chickens. The activities of ALT and ALP may also increase in the serum if there is cellular injury in liver or muscle caused by excessive stress [58]. In this context, probiotics and prebiotics have been reported to reduce stress in hens [41, 42].

Heterophils are one of the abundant granulocytes in most avian species. They perform phagocytosis, have bactericidal properties, and play important roles in acute inflammation [60]. Heterophil numbers usually increase during mildly or moderately stressful conditions, and consequently, the H/L ratio can be used to detect the presence of physiological stress in chickens [61]. Davis et al. [62] showed that the H/L ratio tended to increase when the laying hens were under stressful conditions. In the current study, the inclusion of PRE, PRO or SYN in hen diets decreased the percentage of heterophils and increased the percentage of lymphocytes, leading to a decrease in the H/L ratio compared with those of the control hens. Similar findings were reported by Khan et al. [63] who found that multi-strain probiotic (Protexin®) supplementation in HyLine hens increased the percentage of lymphocytes, and decreased the percentage of heterophils and the H/L ratio. Kim et al. [64] also observed an increase in the percentage of lymphocytes and a decrease in the H/L ratio in broiler chickens supplemented with mannan-oligosaccharide (MOS). Lymphocytes play an important role in humoral antibody formation and cellular immunity. The increase in lymphocyte percentages observed in the PRE, PRO or SYN treatment group indicates an immunostimulatory effect. Probiotics and prebiotics have been reported to stimulate a protective immune response and improve resistance to microbial pathogens in broiler chickens and laying hens [47, 65]. Thus, the results obtained from this study indicate that the supplementation of PRE, PRO or SYN could reduce the stressful effects and stimulate the immune response of laying hens throughout the experimental period.

In the present study, there were no significant differences between the supplemented and control groups in minerals and electrolytes (serum Ca, P, Na, K, Cl), kidney function indicator (uric acid), total protein, glucose, haemogram (RBC, Hb, MCV, MCHC, PCV), leukogram (monocyte, eosinophil and basophil) or thrombocyte count, and all of these parameters were within the normal ranges [66, 67]. It was concluded that the supplementation of the prebiotic IMO, the probiotic PrimaLac® and their combination as a synbiotic did not have any adverse effects on the mineral and electrolyte balances, kidney function, protein and glucose metabolisms, erythrocyte and leucocyte morphology and functions, or blood coagulation homeostasis of thrombocytes in laying hens at 36 and 52 weeks of age.

The relative weights of the heart, liver, ovary, pancreas and spleen were not affected by the incorporation of PRE, PRO or SYN in the diets of laying hens, indicating that the three feed supplements did not exhibit any adverse effect on the internal organ functions of the laying hens at 36 and 52 weeks of age. These results are similar to those reported by Abdel-Raheem and Abd-Allah [33] who observed that feeding MOS, *Saccharomyces cerevisiae* and their combination as a synbiotic had no effect on the weights of the hearts and livers of broiler chickens. Ashayerizadeh et al. [68] also reported that the weights of the heart, liver and pancreas were not significantly different in broiler chickens supplemented with prebiotic (Biolex®- MB), probiotic (PrimaLac®) and their mixture as a synbiotic.

## Conclusions

The results from the present study demonstrated the beneficial effects of PRE (prebiotic IMO), PRO (probiotic PrimaLac®) and SYN (IMO + PrimaLac®) supplementations in laying hens. The inclusion of PRE, PRO and SYN in hen diets improved the feed intake and egg production from 20 to 36 weeks of age, and body weight gain, FCR, egg weight, egg mass and egg size from 20 to 52 weeks of age. Hens fed with PRE, PRO or SYN diet had also reduced the levels of serum total cholesterol, LDL cholesterol, ALP, ALT, heterophil percentage and H/L ratio, and increased the lymphocyte percentage at 36 and 52 weeks of age. Furthermore, the supplementation of PRE, PRO and SYN did not have adverse effects on the weight of vital organs such as the heart, liver, ovary, pancreas and spleen of laying hens at 36 and 52 weeks of age. The results indicate that PRE, PRO or SYN could be used as alternatives to AGPs for improving the health and productivity of laying hens. Considering the ability of PRO diet to improve hen performance at lower feed cost in the present study, PRO diet alone is the best among the three supplementation diets to be recommended for use in commercial layer production for maximum profitability.

## Abbreviations

AGPs: Antibiotic growth promoters
ALP: Alkaline phosphatase
ALT: Alanine aminotransferase
AST: Aspartate aminotransferase
BSH: Bile salt hydrolase
CK: Creatinine kinase
FCR: Feed conversion ratio
FOS: Fructooligosaccharide
GGT: Gamma-glutamyl transpeptidase
H/L: Heterophil to lymphocyte ratio
Hb: Haemoglobin
HDL: High-density lipoprotein
IMO: Isomaltooligosaccharide
K_2_EDTA: Potassium ethylene diamine tetraacetic acid
L: Large
LDL: Low-density lipoprotein
M: Medium
MCHC: Mean corpuscular haemoglobin concentration
MCV: Mean corpuscular volume
MOS: Mannan-oligosaccharide
NCR: National Research Council
PCV: Packed cell volume
PRE: Basal diet +1% isomaltooligosaccharide
PRO: Basal diet +0.1% PrimaLac®
RBC: Red blood cell
S: Small
SCFA: Short chain fatty acid
SPSS: Statistical Package for the Social Sciences
SYN: Basal diet +1% isomaltooligosaccharide +0.1% PrimaLac®
USDA: United States Department of Agriculture
VLDL: Very-low-density lipoprotein
XL: Extra large
